# Comparing growth patterns of three species: Similarities and differences

**DOI:** 10.1371/journal.pone.0224168

**Published:** 2019-10-23

**Authors:** Norbert Brunner, Manfred Kühleitner, Werner Georg Nowak, Katharina Renner-Martin, Klaus Scheicher

**Affiliations:** University of Natural Resources and Life Sciences, Department of Integrative Biology and Biodiversity Research, Vienna, Austria; State Museum of Natural History, GERMANY

## Abstract

Quantitative studies of the growth of dinosaurs have made comparisons with modern animals possible. Therefore, it is meaningful to ask, if extinct dinosaurs grew faster than modern animals, e.g. birds (modern dinosaurs) and reptiles. However, past studies relied on only a few growth models. If these models were false, what about the conclusions? This paper fits growth data to a more comprehensive class of models, defined by the von Bertalanffy-Pütter (BP) differential equation. Applied to data about *Tenontosaurus tilletti*, *Alligator mississippiensis* and the Athens Canadian Random Bred strain of *Gallus gallus domesticus* the best fitting growth curves did barely differ, if they were rescaled for size and lifespan. A difference could be discerned, if time was rescaled for the age at the inception point (maximal growth) or if the percentual growth was compared.

## Introduction

Mathematical growth models aim at a simplified description of growth in terms of curves that fit well to size-at-age data [1]. As the growth of animals depends on multiple factors, the most-informative data came from controlled studies, e.g. for chicken [2] or for pigs [3]. By contrast, for wildlife and wild-caught fish, there remained considerable uncertainties about the proper choice of the growth model [4]. Dealing with extinct species the situation was even worse, as no weighing of body mass was possible for fossils. Nevertheless (e.g. Table 1), recent approaches led to mathematical growth models for dinosaurs [5] that have “revolutionized our understanding of dinosaur biology” [6]. For instance, it is now consensus that dinosaurs grew faster than modern reptiles.

**Table 1:** Age and mass data of Tenonotosaurus tilletti from [18].

age, yr	1	2	2	7	8	8	8	10	11	12	22	26
mass, kg	23	45	61	336	389	560	646	628	306	843	964	1102

**Fig 1:**
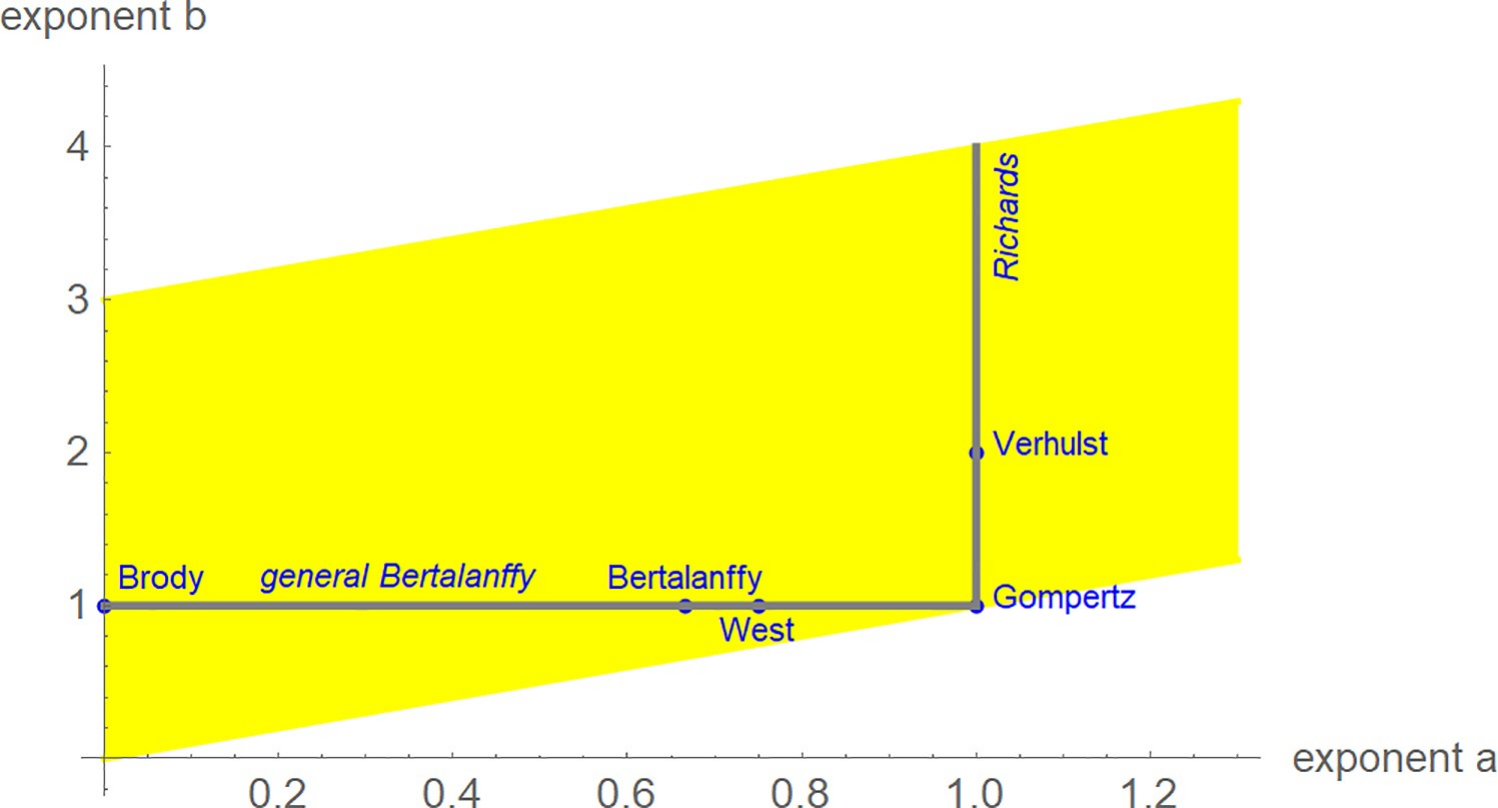
Named models (blue) and part of the search-region (yellow) for the exponent-pair of the best fitting growth model.

However, previous growth studies relied on few models only, whence model uncertainty may be an issue for the comparisons of growth curves of different species. (This paper compares several thousand models, as outlined in Fig 1). Another issue is scaling-up. [7] defined dimensionless mass and time ratios and concluded from a plot that the so rescaled growth data of 13 species were close to a “universal growth curve”. Thus, aside from the different scaling, all animals would grow in the same way. We therefore reconsider the conceptual question, how to compare the growth of species that differ in size and life span.

With respect to model uncertainty, this paper studies a differential Eq (1) of [8], that includes e.g. the models of Brody [9], von Bertalanffy [10], or Verhulst [11] as special cases. In view of its generality it allows a more accurate comparison of growth curves. Literature refers to this model as Bertalanffy-Pütter (BP) model; c.f. [12].

$$m´(t)=p∙{m(t)}^{a}-q∙{m(t)}^{b}$$ (1)

Eq (1) describes growth of mass *m*(*t*) at time *t* and it uses five free parameters that are optimized to obtain a growth curve with a best fit to given data. [13] related the non-negative exponent-pair *a* <*b* to the metabolism. The non-negative constants *p* and *q* are scaling constants. The fifth parameter is the initial condition of the differential equation that is needed to determine the function *m*(*t*); e.g. *m*(0) = *m*_0_, where *m*_0_ > 0 is an estimate for the hatching (or natal) mass.

In this paper we interpret Eq (1) as a definition for a class of models; the BP-class. Thereby, each exponent-pair defines a unique BP-model. For instance, the Verhulst-model is defined from the exponent-pair *a* = 1, *b* = 2. Each BP-models has three free parameters (*p*, *q* and *m*_0_). Fig 1 illustrates this approach and it indicates the exceptional character of these named models, when compared to the range of possible models, whereby for this paper we confined the search for the best-fit exponent-pairs to the yellow area. Richards’ model [14], and the generalized Bertalanffy model of Pauly [15] are represented as line segments (i.e. subclasses of the class of BP-models). Further, the model of Gompertz [16] in the following sense is a limit-case of the BP-class: Growth-curves of the Gompertz model are limits, for (*a*, *b*) → (1, 1), of BP-growth curves with exponents *a*, *b* [17].

Lee and Werning [18] compared the growth of *Tenontosaurus tilletti* with the growth of modern *Alligator mississippiensis* and they concluded that dinosaurs (more specifically: iguanodontids) were not scaled-up lizards, as they grew much faster. We revisit this issue and seek the best fitting BP-models for their data. These data are from [18] (Table 2) about *Tenontosaurus tilletti* (twelve data points, mass 23–1102 kg, age 1–26 years) and the data about *Alligator mississippiensis* (41 data points, mass 0.1–40.7 kg, age 1–42 years), which we retrieved from a plot in [18] (using DigitizeIt of Bormisoft^®^).

**Table 2:** Parameters of the best-fitting models.

Species:	chicken	alligators	tenontosaurs
Exponent *a*:	0.89	0.68	0.8
Exponent *b*:	0.93	0.85	0.9
Initial condition *m*_0_:	32.92 g	158.82 g	22.18 kg
Scaling parameter *p*:	1.0952	1.6843	6.3743
Scaling parameter *q*:	0.7988	0.8882	3.1769
Asymptotic mass *m*_*max*_:	2.67 kg	43.12 kg	1057.5 kg
Full age *t*_*full*_:	184 d	36 a	21 a
Maximal growth rate $m_{max}^{\prime }$ (inflection point):	7.3 kg/a	1.78 kg/a	72.5 kg/a
Mass at the inflection point *m*_*infl*_:	890 g	11.6 kg	325.7 kg
Age at the inflection point *t*_*infl*_:	61 d	9.85 a	6.37 a

Note: We used the initial condition *m*(*t*_*first*_) = *m*_0_, where *t*_*first*_ was the first age of the data.

We also verified the alligator-data from the original source [19], who over a time span of forty years captured and partly recaptured ca. 7000 alligators from Louisiana, USA. In order to explore the limits of dinosaur growth, we used data about modern avian dinosaurs, broiler chicken that were bred for fast growth and reared under optimal conditions. To this end we identified the best fitting BP-model for the data from [2] (Table 1) about the Athens Canadian Random Bred strain of *Gallus gallus domesticus* (28 data points, mass 0.04–2.23 kg, age 0–170 days).

Within the BP-class, model uncertainty was related to the variability of the exponents. To this end, the paper identified the region of near-optimal exponent-pairs. The exponent-pairs of this region could also be used to model growth without affecting the fit to the data significantly when the other parameters were optimized. We used them to explore the model uncertainty.

The best-fitting and the near optimal exponents were then used to compare the growth of different species despite their different scales in size and age. In addition, a dinosaur-year had more days, but these were shorter. As overall a year covered about the same time span as today, we used kg and years as units; e.g. weight gains in kg/year also for chicken.

## Methods

The methods are explained in detail in our preprint [20] at BioRxiv. We therefore point out only the main issues. As was observed e.g. for chicken [2], the standard deviation of mass becomes higher for heavier animals, whence the method of least squares may not be suitable for data-fitting. Instead, as in [21] we minimized the sum of squared errors between the logarithm of the growth function and the logarithmically transformed data (*SSLE*). This defined the following function (2):

$${SSLE}_{opt}(a,b)=\min_{m_{0},p,q}\left(SSLE\right)$$ (2),

assuming model (1) with exponents *a*, *b*. An exponent-pair was near-optimal, if its *SSLE*_*opt*_(*a*, *b*) exceeded the least *SSLE* by less than 5%. We did not minimize *SSLE* for each exponent-pair. Instead we considered exponent pairs of the search region (yellow area in Fig 1) on a grid (distance 0.01 in the *x* and *y* directions, respectively). Thereby, we searched 26,200 grid-points for the chicken, 88,730 for the alligators and 42,371 for *Tenontosaurus*.

## Results

Table 2 summarizes the model parameters that minimized *SSLE*. The parameters for chicken are from [21]. In order to define dimensionless coordinates, asymptotic mass *m*_*max*_ was computed as the limit of the growth curve *m*(*t*), when time *t* approaches infinity. At “full age”, *t*_*full*_, 90% of the asymptotic mass were reached; we used “full age” as a proxy for “adulthood”. (Further, we used 90% to avoid excessive extrapolations, if the asymptotic mass was larger than the observations.) The inflection point is defined by the maximal growth rate $m_{max}^{\prime }$; it was attained at age *t*_*infl*_ with mass *m*_*infl*_. In comparisons between species the maximal growth rate (i.e. *m´*(*t*_*infl*_)) is used as a proxy for the basal metabolic rate [22]. These parameters were all computed from the best fitting model.

In order to compare the growth curves, they were rescaled in dimensionless coordinates. Ideally, the dimensionless time coordinate corresponds to about the same stage of the biological development of the considered animals. We use a linear rescaling, assuming *t* = 0 has the same biological meaning for the considered animals and seeking a second point of time with the same meaning. Fig 2 of [20] used full age *t*_*full*_; i.e. mass was reported as a fraction of the asymptotic mass (*m*_*max*_) and time was expressed as a fraction of full age. In terms of these dimensionless coordinates, the best-fitting model curves were almost equal. The rescaled data, too, were close to these curves, except for a larger spread for tenontosaurs. Similar plots were obtained, if instead of *t*_*full*_ the half-weight age was used (i.e. *m*(*t*) = *m*_*max*_/2) or any other fraction of the asymptotic weight (e.g. 15%). As this seemed to indicate that all animals would grow alike, aside from rescaling, we checked also other empirically meaningful ages.

**Fig 2:**
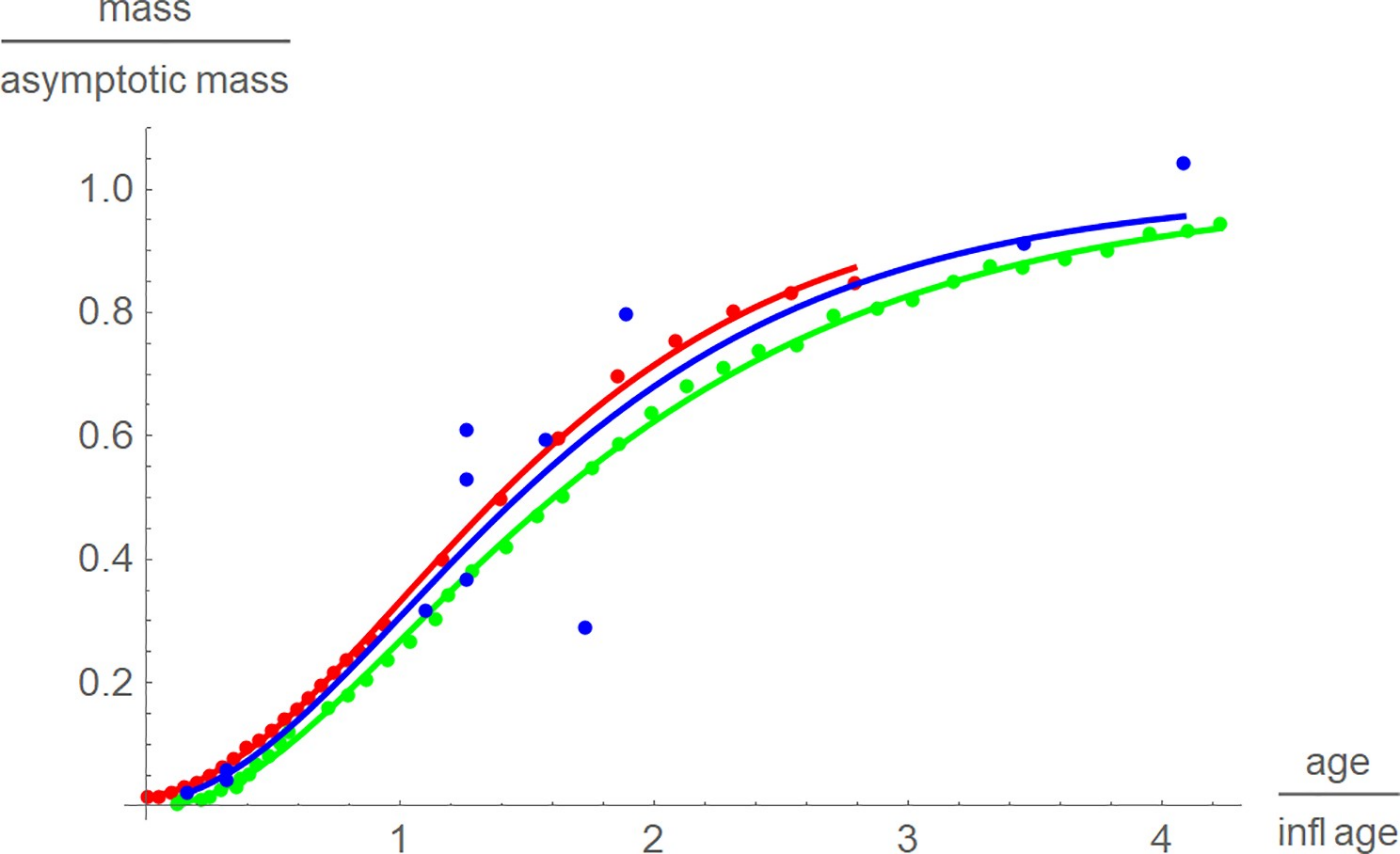
Growth data and best fitting growth curves in dimensionless coordinates (mass as a fraction of *m*_*max*_, time as a fraction of *t*_*infl*_) for chicken (red), alligators (green), and tenontosaurs (blue).

In Fig 2 we used *t*_*infl*_. In terms of this rescaling, a difference between the species could be discerned, although the rescaled growth curves remained close together: The graphical representation of the results uses red for chicken, green for alligators and blue for *Tenontosaurus*. Chicken grew faster than tenontosaurs, and these grew faster than alligators, whereby some tenontosaurs (blue points) grew even faster than chicken and slower than alligators.

**Fig 3:**
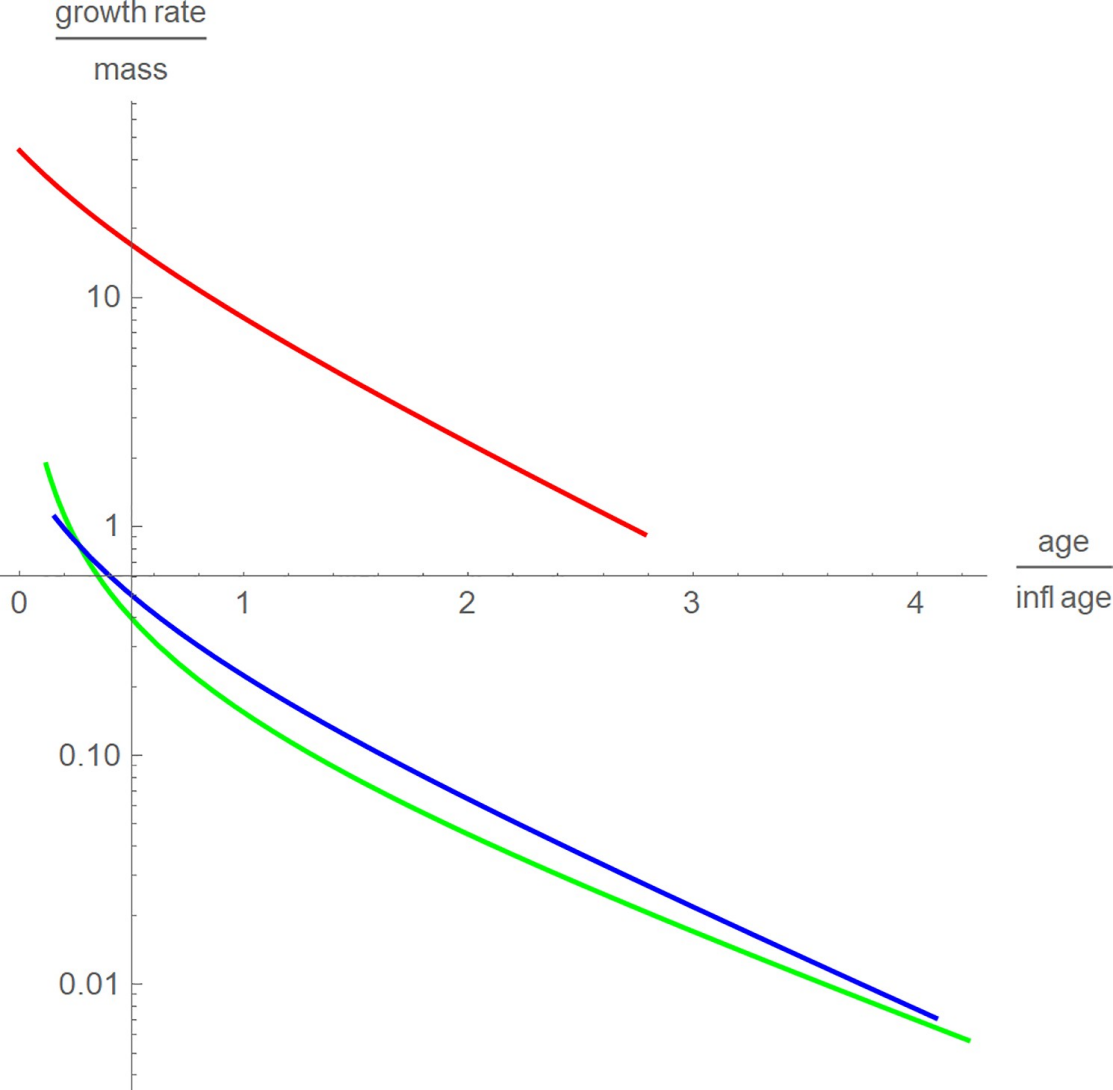
Growth rates relative to body mass for chicken (red), alligators (green) and tenontosaurs (blue) with time as a fraction of *t*_*infl*_.

As for another comparison of the growth, in Fig 3 we compared the relative growth rates *m´*(*t*)/*m*(*t*) using a dimensionless time scale. This graphical representation emphasized the differences in the growth rates best: well-fed broiler chicken grew more than ten times faster than alligators and *Tenontosaurus* and the latter grew somewhat faster than alligators. Fig 3 displays this for the rescaling using *t*_*infl*_. In [20] this was also observed using *t*_*full*_ for rescaling.

**Fig 4:**
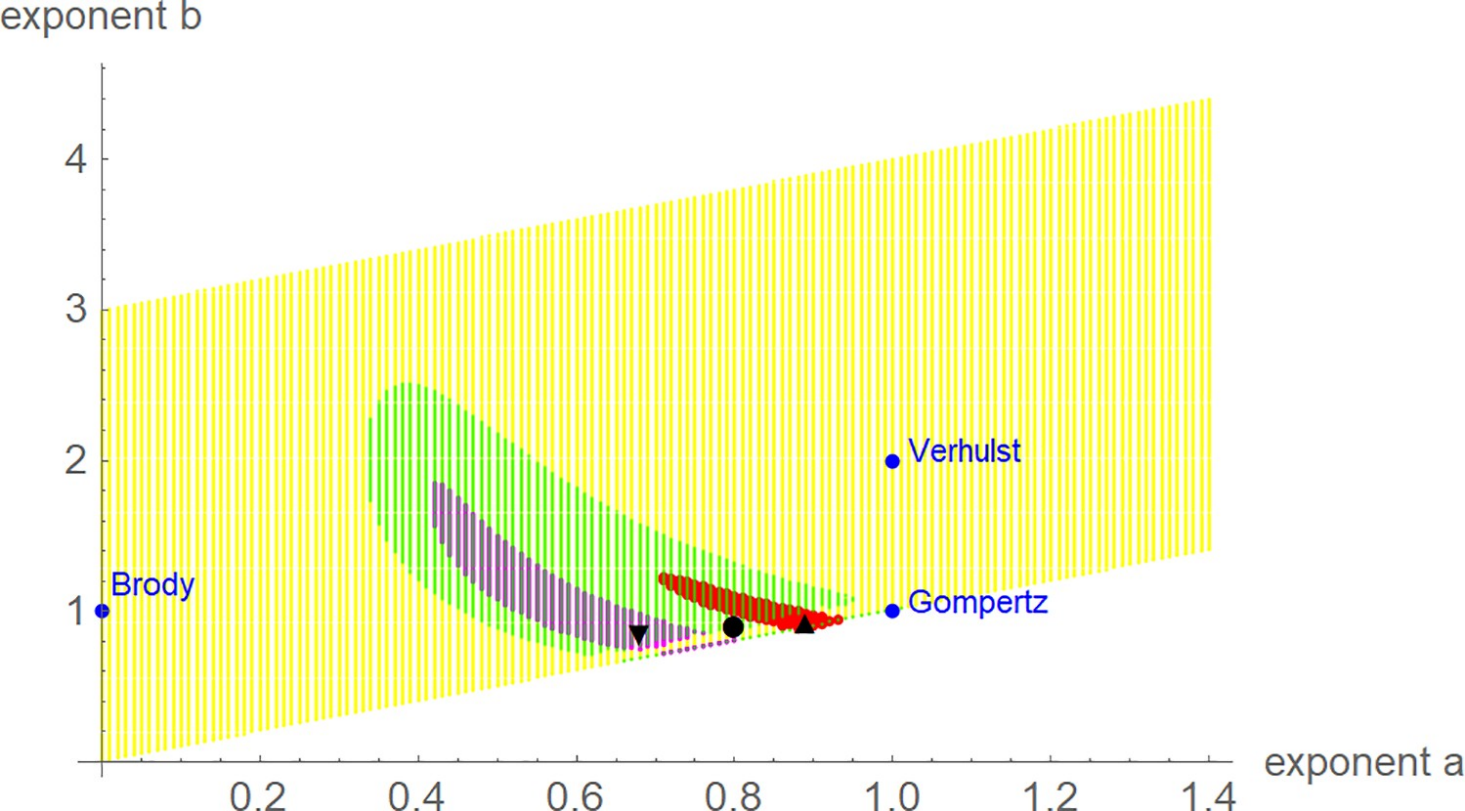
Optimal and near-optimal exponent-pairs for chicken (triangle and red area), alligators (upside triangle and green area) and tenontosaurs (circle and blue area). For better orientation, the exponent-pairs of three named models are plotted (blue).

With respect to model uncertainty, Fig 4 plots the optimal and near-optimal exponent-pairs. Despite the similarity of the data in dimensionless coordinates, the optimal exponent-pairs were different. However, due to the larger variance of the dinosaur-data the region of near-optimal exponents for dinosaurs was larger and it included the regions for alligators and for chicken. Thus, judging from the perspective of extinct dinosaurs, their growth data did not display a systematic difference to modern species, whence there was no fundamental change in the growth pattern. The regions of near-optimal exponents displayed fuzzy boundaries and points close to the diagonal were not connected to the regions. This was caused by the optimization strategy, aiming at a high accuracy for points next to the diagonal and at faster computations thereafter. However, despite these deficiencies the visualization of the near-optimal exponents verified the optimal character of the optimal exponent-pairs.

**Fig 5:**
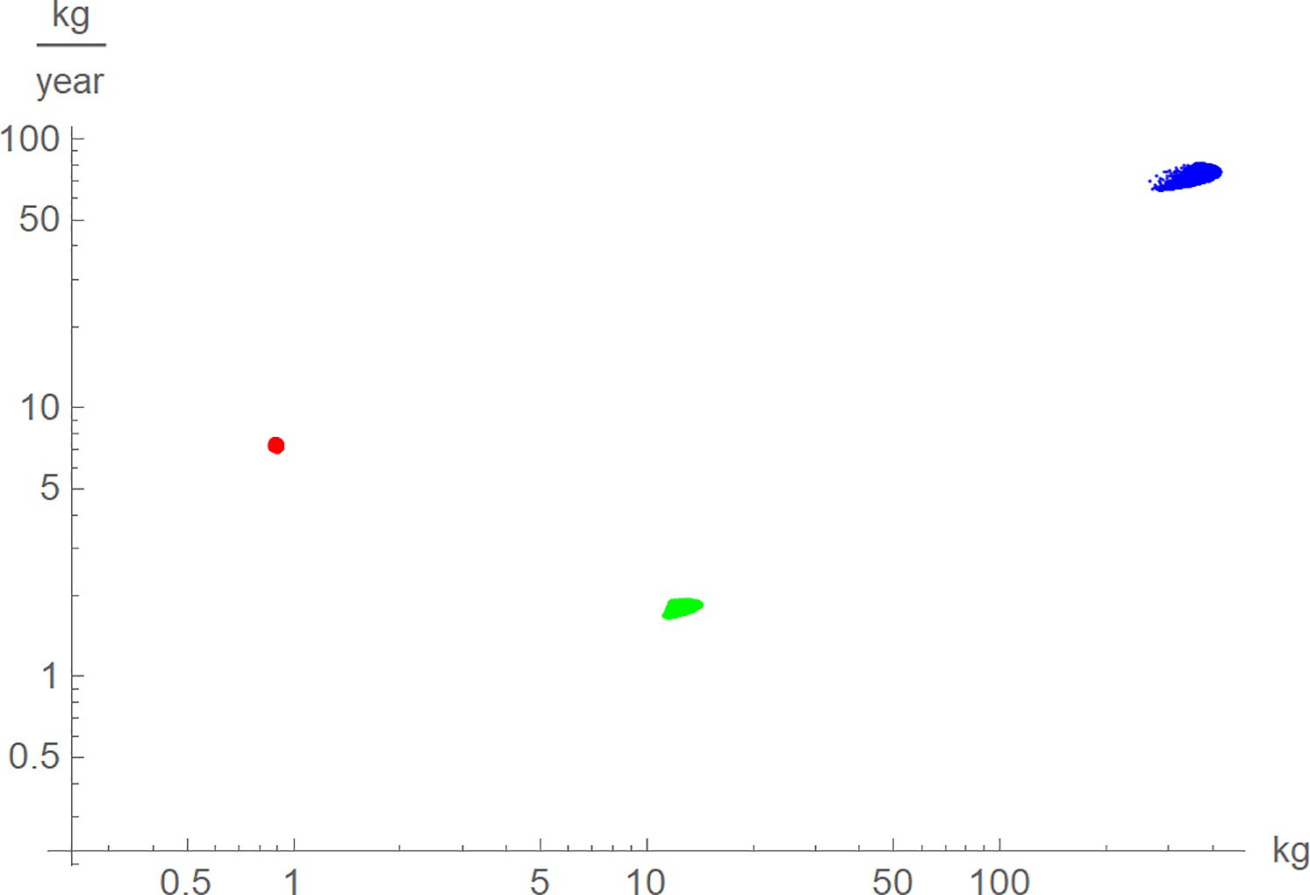
Maximal growth rate, *m´*(*t*_*infl*_), and mass at the inflection point, *m*_*infl*_ = *m*(*t*_*infl*_), for near-optimal growth curves *m*(*t*) for chicken (red), alligators (green) and tenentosaurs (blue).

Fig 5 used the near-optimal models to explore, how sensitive the maximal growth rate was to the choice of a model. The growth rate is a measure that cannot be observed directly from the data; it is derived from a growth model and depends on what model is selected. This was demonstrated for the maximal growth rate, which varied considerably even for growth curves that fitted well to the data. The clouds in Fig 5 display the values of *m* and *m´* at the inflection point of *m*(*t*), using near-optimal growth curves. Apparently, even well-fitting growth curves resulted in inaccurate estimates for the maximal *m´*. Nevertheless, regardless of the near-optimal model used, *m´*(*t*_*infl*_) for chicken was much higher than *m´*(*t*_*infl*_) for the larger alligators, and *m´*(*t*_*infl*_) for dinosaurs was largest, whereas relative to body size, i.e. in terms of *m´*(*t*_*infl*_)/*m*(*t*_*infl*_), chicken grew fastest.

## Discussion

For the data about three species of dinosaurs from [18] only *Tenontosaurus* provided feasible data. For the two other species, the plot of the near-optimal exponent-pairs (c.f. Fig 4) displayed large regions that almost covered the search grid. As a large region of near-optimal exponents indicates that data may not carry enough information to differentiate between growth models, the paper did not use them. However, in view of the inherent uncertainties of estimating the mass of dinosaurs [6], it was surprising that one in three datasets allowed to differentiate between the models.

Further, for all species the optimal exponent-pairs were quite remote from the exponent-pairs for the named models which are more common in growth studies. This indicates that BP-models provide a significantly better fit than the conventionally used models. In fish-biology it has long been accepted that exponent-pairs (*a*, *b*) with *a* < 1 and *b* = 1 might be better compatible with biological constraints for growth; e.g. the growth of gill surface area relative to mass growth [16]. Recently, also exponents *b* < 1 were considered as biologically meaningful [23]. In epidemiology, too, recent publications supported the use of BP-models to analyze outbreaks of diseases, e.g. [24].

The issue of rescaling proved to be tricky. Using the “full age” to define dimensionless coordinates did not allow to discern different growth patterns for different species. Using the age at the inflection point was more satisfactory and this age may have a biological meaning (phenomenologically, growth is fastest at this age). For the present data, this rescaling resulted in the expected outcome: Broiler chicken grew fastest and *Tenontosaurus* grew faster than modern reptiles. This pattern was confirmed under three different perspectives (Figs 2, 3 and 5). However, using a linear transformation for rescaling may be an oversimplification, as for different species the fraction *t*/*t*_*infl*_ may correspond to different stages of their biological development. Yet, using this linear transformation was a convenient tool to combine data and growth curves into one plot. Further, with respect to Fig 4 the faster growth of broiler chicken will also be observed for any nonlinear transformation of time that aims at a proper representation of biological development.

## Conclusion

It is generally acknowledged that mass-at-age estimates for dinosaurs are highly uncertain. It was therefore surprising that data for *Tenontosaurus* allowed for the identification of a best fitting growth model within the comprehensive class of BP-models (1) with relatively low variability in the parameters (i.e. small region of the near-optimal exponent-pairs). However, data uncertainty did not allow to conclude that *Tenontosaurus* would need a different exponent-pair (model) than modern alligators or birds. On the contrary, depending on the rescaling, displaying the data in dimensionless coordinates did not always show notable differences. In order to display differences, we rescaled mass relative to the asymptotic limit (of the best fitting model) and time relative to the age at the inflection point (age of maximal growth). Using this rescaling, we obtained the expected results: Modern broiler chicken grew much faster than dinosaurs or alligators and dinosaurs grew faster than alligators.

## Supporting information

S1 File Age (years) and mass (kg) data of *Alligator mississippiensis*, retrieved from [18] (Fig 3A). (XLSX)

S2 File Computation of *SSLE*_*opt*_(*a*, *b*), based on S1 File, for certain grid-points, namely exponents *a* and *b*, and for them the best fit-parameters (optimization results) initial mass *m*_*0*_, *p*, *q*, and *SSLE*. (XLSX)

S3 File Age (days) and mass (g) data of male *Gallus gallus domesticus* from [2]. (XLSX)

S4 File Computation of *SSLE*_*opt*_(*a*, *b*), based on S3 File, for certain grid-points, namely exponents *a* and *b*, and for them the best fit-parameters (optimization results) initial mass *m*_*0*_, *p*, *q*, and *SSLE*. (XLSX)

S5 File Age (years) and mass (kg) data of *Tenontosaurus tilleti* from [18]. (XLSX)

S6 File Computation of *SSLE*_*opt*_(*a*, *b*), based on S5 File, for certain grid-points, namely exponents *a* and *b*, and for them the best fit-parameters (optimization results) initial mass *m*_*0*_, *p*, *q*, and *SSLE*. (XLSX)
